# The mediating role of anxiety in disease uncertainty and acute stress in acute ischaemic stroke patients in the post-epidemic era

**DOI:** 10.3389/fpsyt.2023.1218390

**Published:** 2023-10-17

**Authors:** Hui Tan

**Affiliations:** Quzhou TCM Hospital at the Junction of Four Provinces Affiliated to Zhejiang Chinese Medical University/Quzhou Hospital of Traditional Chinese Medicine, Quzhou, China

**Keywords:** anxiety, disease uncertainty, acute stress, acute ischaemic stroke, post-epidemic era

## Abstract

**Objective:**

To analyse the influencing factors of anxiety, disease uncertainty and acute stress response in patients with acute ischaemic stroke, and to verify the mediating role of anxiety in the post-epidemic era.

**Methods:**

240 patients with acute ischaemic stroke were selected from a tertiary hospital in Wuhan City and investigated by questionnaire and convenience sampling methods.

**Results:**

The total anxiety score, disease uncertainty and acute stress reaction were at moderate levels. Anxiety was positively correlated with illness uncertainty, and anxiety and acute stress response were negatively correlated. Multiple linear regression analysis showed that Sickness uncertainty, acute stress response, age, and work status influenced anxiety. Anxiety mediated the prediction of Sickness uncertainty and acute stress response, with the mediating effect accounting for 35.6% of the total effect.

**Conclusion:**

Disease uncertainty in patients with acute ischaemic stroke in the post-epidemic era directly affects the acute stress response and indirectly through anxiety.

## 1. Introduction

Acute ischaemic stroke is a sudden interruption of blood supply to local brain tissue due to various reasons, resulting in necrosis of local brain tissue due to ischemia and hypoxia, followed by symptoms of corresponding neurological deficits (1). There are about 3 million new cases of stroke in China every year. The prevalence of stroke is above 60 years of age, with an average annual increase of 8.3% in the number of first-ever strokes between the ages of 40 and 70 years (2). It is characterised by high morbidity, mortality, disability, and complications, and the incidence and recurrence rates are still high, causing patients to suffer from disease uncertainty, stress, anxiety, and fear, which severely limit their ability to care for themselves and affect their quality of life (3–6). Disease uncertainty is a chronic and pervasive source of psychological distress in patients and plays an important role in stroke recovery (7). Mullins (8, 9) and other related studies have shown that disease uncertainty is a predictor of the development of psychological distress and can be used as a predictor of anxiety. Acute stress is a stress response that is exhibited by an individual between 2 and 28 days after personally experiencing, witnessing, or facing a traumatic event with a threat of death or serious injury to themselves or others (10). Patients with anxiety and depressive symptoms have a more severe acute stress response (11, 12). Anxiety is a common psychological symptom in patients with acute ischaemic stroke (13–20), and Li et al. (21) showed that 16% of patients with acute ischaemic stroke had anxiety. Therefore, the present study explored the correlation between anxiety, disease uncertainty and acute stress response and tested the hypothesis that anxiety has a mediating role in disease uncertainty and acute stress response. The aim was to reduce negative psychology in patients with acute ischaemic stroke in the post-pandemic era. The mediating role in this study refers to whether the effect of illness uncertainty on the acute stress response is examined through the mediating variable anxiety before it affects the acute stress response; that is, whether there is a relationship of illness uncertainty → anxiety → acute stress response, as such.

## 2. Methods

### 2.1. Study design

This study is a cross-sectional study in a non-experimental study.

### 2.2. Study population

The subjects of this study were acute ischaemic stroke patients in a tertiary care hospital in Wuhan, China.

#### 2.2.1. Inclusion criteria

(1) Meeting the diagnostic criteria of the Chinese guidelines for the diagnosis and treatment of acute ischaemic stroke.
(2) Patients with acute ischaemic stroke of first onset.
(3) clear consciousness.

#### 2.2.2. Exclusion criteria

(1) Combination of severe organic lesions of other organs.
(2) Pre-existing psychological and psychiatric disorders.
(3) Unwillingness to cooperate in the middle of the procedure.

#### 2.2.3. Calculation of sample size

The rough estimation method proposed by Li Zheng (22) was used to calculate the sample content, with 12 items of the general demographic data questionnaire, two dimensions of the Illness Uncertainty Scale, five dimensions of the Chinese version of the Stanford Acute Stress Questionnaire, and one dimension of the State Anxiety Scale, for a total of 20 variables, and the sample size was taken as 10 times the number of variables, i.e. the sample size = 20*10*1.2 = 240, and the final sample size was determined to be 240 cases.

### 2.3. Research tools

General Information Questionnaire, Illness Uncertainty Inventory (MUIS-A) (23), Acute Stress Response Questionnaire (SASRQ) (24), State Anxiety Inventory (S-AI) (25).

### 2.4. Theoretical basis

**Figure 1:**
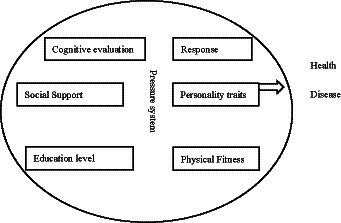
Psychological stress model.

This study is based on the psychological stress model (resilience in process model), and Jiang Qianjin proposed the concept of ‘stress multifactor system’ through the study of multiple factors of psychological stress (26, 27), that is, stress is not a simple cause-effect or stimulus–response process. Rather, it is a multi-factor interaction, feedback regulation and control system composed of stressor, stress response and other related factors, which makes stress a bio-psycho-social integrated system concept (Figure 1).

### 2.5. Research framework

**Figure 2:**
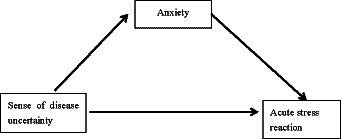
Research framework.

Based on psychological stress theory, this study makes the following hypotheses (1) illness uncertainty has a direct predictive effect on anxiety; (2) illness uncertainty has a direct predictive effect on acute stress response; (3) acute stress response has a direct predictive effect on anxiety; (4) acute stress response has a mediating role in illness uncertainty and anxiety, and the framework of this study is shown in Figure 2.

#### 2.5.1. Neurophysiological mechanisms

anxiety is seen as being most often generated by concurrent and equivalent activation of fear (or frustration) and reward system. If uncertainty disappears, anxiety should give way to satisfaction or joy (if winning) or some negative emotions ranging from sadness to fear. The more active reward system is in a situation of uncertainty, the higher should be anxiety-related arousal (28).

In reinforcement sensitivity theory, Gray proposed several conceptual neural systems, which include: behavioural approach system (BAS), which is a positive behavioural feedback system; behavioural inhibition system (BIS), which is a negative behavioural feedback system; confrontation/flight system (FFS), which has the same function as the behavioural inhibition system but differs from the behavioural inhibition system in that it is more sensitive to conditioned aversions. a negative behavioural feedback system; and the fight/flight system (FFS), which has the same function as the behavioural inhibition system, but differs from the behavioural inhibition system in that it is more sensitive to unconditional aversions and is responsible for the regulation of emotions such as fear and anxiety. Gray et al. (29) revised the three systems in 2000, replacing the confrontation/flight/freeze system (FFFS) with the fight/flight/freeze system (FFFS), which is sensitive to punishments, rewards, and conflicting stimuli, respectively.

### 2.6. Data collection and processing

We communicated with the study subjects and their families, explained the purpose and significance of the study and the method of filling out the questionnaires, and collected the questionnaires on the spot. 320 questionnaires were distributed in this study, 300 were collected, 288 were valid and 12 were invalid, with a valid recovery rate of 96%.

This study used SPSS26.0 for data analysis

(1) The general information of the patients and the count data in the scores of each scale were expressed by frequency (*n*) and percentage (%), and the measurement data were expressed by mean ± standard deviation.
(2) Differences between different patients in terms of scores of each scale were analysed by two independent samples *t*-test and ANOVA.
(3) Pearson’s correlation analysis was used to explore the relationship between anxiety, disease uncertainty, and acute stress response.
(4) Multiple linear regression analysis was used to explore the effects between the three.
(5) ProceSS3.5 macro program was used to further test the mediating effect of anxiety between illness uncertainty and acute stress response.

## 3. Results

### 3.1. General information status in the post-epidemic era

Males accounted for 58.3 percent of the total and females for 41.7 percent. The age distribution was over 50 years old, Han Chinese patients accounted for 64.6 percent, and married patients accounted for 86.7 percent. The place of residence was mostly urban, occupying 76.2%, the cultural level was mainly at junior high school, high school or secondary school level, the working status was mostly retired, occupying 60.4%, the *per capita* monthly income of the family was mostly in the number of people in the range of 2,000-4,000, occupying 54.4, 69.6% of the health care payment method was urban workers’ insurance, the self-assessment of the economic pressure was moderate, and the number of children >2 or ≤ 2 had a small difference. 57.1% had average knowledge of the disease. See Table 1 for details.

**Table 1:** General information status of study subjects (*n* = 240, %).

Item	Group	Number of cases	Composition ratio
Gender	Male		
	Female	140	58.3
Age (years)	①<60	100	41.7
	②60 ~	72	30.0
	③ ≥ 70	87	36.3
Ethnicity	Han Chinese	81	33.7
	Ethnic Minority	155	64.6
Marital status	With spouse	85	35.4
	No spouse	32	13.3
Education level	①Elementary school and below	208	86.7
	②Junior high school	55	22.9
	③High school or junior high school	88	36.7
	④College and above	73	30.4
Work status	①Working	24	10.0
	②retired	33	13.8
	③Unemployed	145	60.4
Place of residence	Urban	62	25.8
	Rural	183	76.2
*Per capita* monthly income	①<2,000	57	23.8
(Yuan)	②2,000 ~	69	28.8
	③ ≥ 4,000	131	54.5
		40	16.7
Medical payment method	①Town insurance	167	69.6
	②Urban and rural insurance	48	20.0
	③Self-paid	25	10.4
Self-assessed financial pressure	①No or small	61	25.4
	②Medium	106	44.2
	③Larger	73	30.4
Number of children	≤2	115	47.9
	>2	125	52.1
Degree of disease understanding	①Very well	14	5.8
	②General	137	57.1
	③ Do not know	89	37.1

### 3.2. Status of disease uncertainty in the post-epidemic era

#### 3.2.1. Disease uncertainty score in the post-epidemic era

Compared to the complexity dimension scores, the uncertainty dimension scores were high with a total score (48.59 ± 4.96) and the total disease uncertainty score (77.64 ± 6.89), 98.3% of the patients had a moderate level of disease uncertainty. Only 1.7% of patients were at a high level. The details are shown in Tables 2, 3.

**Table 2:** Total disease uncertainty scores and scores of each dimension in the study subjects (*n* = 240, $\bar{x}$ ± *s*).

Variables	Number of entries Score	Score range	Total score	Average score of entries
Uncertainty Dimension	15	15 ~ 75	48.59 ± 4.96	3.24 ± 0.33
Complexity dimension	10	10 ~ 50	29.05 ± 3.12	2.91 ± 0.31
Total disease uncertainty score	25	25 ~ 125	77.64 ± 6.89	3.11 ± 0.28

**Table 3:** Status of disease uncertainty level in study subjects (*n* = 240, %).

Variables	Classification	Score range	Number of examples	Composition ratio
Sense of disease uncertainty	Low level	25.0 ~ 58.3	0	0.0
	Medium level	58.4 ~ 91.7	236	98.3
	High level	91.8 ~ 125	4	1.7

#### 3.2.2. Single-factor analysis of disease uncertainty in the post-epidemic era

Age in the 60–70 years old disease uncertainty scored the highest score of 79.00 ± 5.86 points, ethnic minorities scored 79.04 ± 7.09 points, patients without spouses scored 79.04 ± 7.09 points, literacy in primary school culture scored 79.67 ± 6.00 points, joblessness scored 80.40 ± 7.10 points, most of the place of residence in rural areas, *per capita* income per month is less than 2,000 yuan, and most of them are self-paying patients with a score of 81.36 ± 5.73 points, self-assessed economic pressure, the difference in the number of children is small, and the patients do not understand their own diseases, with a score of 80.62 ± 6.41 points, see Table 4 for details.

**Table 4:** Univariate analysis of uncertainty of disease (*n* = 240, $\bar{x}$ ± *s*).

Item	Group	Score	*t*/F	*P*	*LSD*
Gender	Male	76.99 ± 7.16	−1.742	0.083	
	Female	78.55 ± 6.41			
Age (years)	①<60	75.50 ± 7.51	5.215	0.006	①<②
	②60 ~	79.00 ± 5.86			
	③ ≥ 70	78.07 ± 6.96			
Ethnicity	Han Chinese	76.87 ± 6.67	−2.350	0.020	
	Ethnic Minority	79.04 ± 7.09			
Marital status	With spouse	77.02 ± 6.61	3.605	<0.001	
	No spouse	81.63 ± 7.41			
Education level	①Elementary school and below	79.67 ± 6.00	3.427	0.018	①>③④
	②Junior high school	77.99 ± 6.99			
	③High school or junior high school	76.45 ± 6.99			
	④College and above	75.29 ± 7.09			
Work status	①Working	73.03 ± 6.69	13.731	<0.001	①<②<③
	②retired	77.50 ± 6.26			
	③Unemployed	80.40 ± 7.10			
Place of residence	Urban	76.75 ± 6.43	−3.651	<0.001	
	Rural	80.47 ± 7.56			
*Per capita* monthly income	①<2,000	80.32 ± 6.70	15.137	<0.001	①>②>③
(Yuan)	②2,000 ~	77.58 ± 6.33			
	③ ≥ 4,000	73.20 ± 6.79			
Medical payment method	①Town insurance	76.71 ± 6.98	6.299	0.002	①<②③
	②Urban and rural insurance	78.94 ± 6.33			
	③Self-paid	81.36 ± 5.73			
Self-assessed financial pressure	①No or small	75.90 ± 6.81	9.223	<0.001	①②<③
	②Medium	76.75 ± 6.62			
	③Larger	80.38 ± 6.60			
Number of children	≤2	76.31 ± 7.16	−1.917	0.057	
	>2	78.46 ± 6.27			
Degree of disease understanding	①Very well	71.50 ± 6.10	18.805	<0.001	①<②<③
	②General	76.33 ± 6.46			
	③ Do not know	80.62 ± 6.41			

### 3.3. Acute stress response status in the post-epidemic era

#### 3.3.1. Acute stress response score in the post-epidemic era

The scores for each dimension were as follows, dissociative symptoms (24.69 ± 6.67) with the highest score, hypervigilant symptoms (17.71 ± 5.12), avoidance symptoms (15.23 ± 4.53), re-experiencing symptoms (15.63 ± 4.39), impairment of social functioning (4.54 ± 1.64) with the lowest score, and the total score of acute stress reaction (77.80 ± 20.29) points, detailed results are shown in Tables 5, 6.

**Table 5:** Total score of acute stress response and scores of each dimension in the study subjects (*n* = 240, $\bar{x}$ ± *s*).

Variables	Number of entries	Score range	Total score	Average score of entries
Dissociative symptoms	10	0 ~ 50	24.69 ± 6.67	2.47 ± 0.67
Hypervigilance symptoms	6	0 ~ 30	17.71 ± 5.12	2.95 ± 0.85
Avoidance symptoms	6	0 ~ 30	15.23 ± 4.53	2.54 ± 0.75
Recurrent symptoms	6	0 ~ 30	15.63 ± 4.39	2.60 ± 0.73
Impairment of social functioning	2	0 ~ 10	4.54 ± 1.64	2.27 ± 0.82
Total Acute Stress Score	30	0 ~ 150	77.80 ± 20.29	2.59 ± 0.68

**Table 6:** Levels of acute stress in study subjects (*n* = 240, %).

Variables	Classification	Score range	Number of cases	Composition ratio
	None	<40	7	2.9
Acute stress response	Moderate	40 ~ 56	194	80.8
	Severe	57 ~ 150	39	16.3

#### 3.3.2. Univariate analysis of acute stress response in the post-epidemic era

Age ≥ 70 years of age had the highest acute stress score of 82.90 ± 16.19 points, patients without spouses scored 84.97 ± 22.45 points, literacy in primary school culture scored 85.95 ± 14.48 points, joblessness scored 83.35 ± 18.34 points, the *per capita* monthly income was less than 2,000 yuan, scored 82.86 ± 18.48 points, and most of them were self-funded patients, self-assessed economic pressure, the difference in the number of children is small, patients do not understand their own disease, score 82.56 ± 18.64 points, see Table 7.

**Table 7:** Univariate analysis of acute stress response (*n* = 240, $\bar{x}$ ± *s*).

Item	Group	Score	*t*/F	*P*	*LSD*
Gender	Male	78.77 ± 21.21	0.877	0.381	
	Female	76.44 ± 18.95			
Age (years)	①<60	73.99 ± 23.68	4.795	0.010	①<③
	②60 ~	76.21 ± 19.96			
	③ ≥ 70	82.90 ± 16.19			
Ethnicity	Han Chinese	76.08 ± 20.24	−1.784	0.076	
	Ethnic Minority	80.94 ± 20.14			
Marital status	With spouse	76.70 ± 19.77	2.163	0.032	
	No spouse	84.97 ± 22.45			
Education level	①Elementary school and below	85.95 ± 14.48	5.184	0.002	①>②③④
	②Junior high school	77.35 ± 20.83			
	③High school or junior high school	74.84 ± 21.82			
	④College and above	69.79 ± 20.00			
Work status	①Working	69.36 ± 23.76	4.815	0.011	①<③
	②retired	77.34 ± 19.64			
	③Unemployed	83.35 ± 18.34			
Place of residence	Urban	76.51 ± 20.79	−1.897	0.061	
	Rural	81.93 ± 18.17			
*Per capita* monthly income	①<2,000	82.86 ± 18.48	3.900	0.022	①>②③
(Yuan)	②2,000 ~	76.85 ± 20.68			
	③ ≥ 4,000	72.20 ± 20.55			
Medical payment method	①Town insurance	76.05 ± 21.21	5.545	0.005	①②<③
	②Urban and rural insurance	78.52 ± 18.68			
	③Self-paid	88.08 ± 13.23			
Self-assessed financial pressure	①No or small	69.08 ± 17.36	15.781	<0.001	①<②<③
	②Medium	77.21 ± 21.68			
	③Larger	85.95 ± 17.26			
Number of children	≤2	75.17 ± 21.69	−1.928	0.055	
	>2	80.22 ± 18.67			
Degree of disease understanding	①Very well	63.07 ± 15.32	6.880	0.001	①<②③
	②General	76.21 ± 20.90			
	③ Do not know	82.56 ± 18.64			

### 3.4. Anxiety status in the post-epidemic era

#### 3.4.1. Anxiety score situation in the post-epidemic era

Thirty four were patients with no anxiety (14.2%), 99 with mild anxiety (41.3%). Ninety one with moderate anxiety (37.9%). Sixteen patients with severe anxiety (6.6%) accounted for a smaller percentage. See Table 8 for details.

**Table 8:** Anxiety scores of the study participants (*n* = 240, %).

Variables	Classification	Score range	Number of cases	Composition ratio
Anxiety	None	<50	34	14.2
	Mild anxiety	50 ~ 59	99	41.3
	Moderate anxiety	60 ~ 69	91	37.9
	Severe anxiety	70 ~ 80	16	6.6

#### 3.4.2. Univariate analysis of anxiety in the post-epidemic era

Age ≥ 70 years old had the highest anxiety score, 60.83 ± 6.64 points, literacy in primary school education score 61.98 ± 6.51 points, jobless status score 61.02 ± 8.03 points, and most of them were self-paying patients with a score of 61.92 ± 6.32 points, and self-assessment of economic stress was high, 61.21 ± 7.37 points. The difference in the number of children was small, and the patients were unaware of their disease, scoring 59.91 ± 7.72 points, as shown in Table 9.

**Table 9:** Univariate analysis of anxiety (*n* = 240, $\bar{x}$ ± *s*).

Item	Group	Score	*t*/F	*P*	*LSD*
Gender	Male	58.20 ± 8.05	−0.814	0.417	
	Female	58.99 ± 6.92			
Age (years)	①<60	56.00 ± 7.42	8.163	<0.001	①<②<③
	②60 ~	58.48 ± 7.97			
	③ ≥ 70	60.83 ± 6.64			
Ethnicity	Han Chinese	58.33 ± 7.29	−0.550	0.583	
	Ethnic Minority	58.89 ± 8.16			
Marital status	With spouse	58.23 ± 7.19	1.250	0.219	
	No spouse	60.47 ± 9.73			
Education level	①Elementary school and below	61.98 ± 6.51	6.707	<0.001	①>②③④
	②Junior high school	58.16 ± 7.26			
	③High school or junior high school	57.64 ± 7.90			
	④College and above	54.67 ± 7.65			
Work status	①Working	56.21 ± 9.29	4.416	0.016	①②<③
	②retired	57.99 ± 6.71			
	③Unemployed	61.02 ± 8.03			
Place of residence	Urban	57.69 ± 6.98	−2.767	0.007	
	Rural	61.23 ± 8.84			
*Per capita* monthly income	①<2,000	60.36 ± 8.72	2.366	0.100	
(Yuan)	②2,000 ~	57.82 ± 6.68			
	③ ≥ 4,000	57.68 ± 7.99			
Medical payment method	①Town insurance	57.35 ± 7.38	7.154	0.001	①<②③
	②Urban and rural insurance	60.88 ± 7.98			
	③Self-paid	61.92 ± 6.32			
Self-assessed financial pressure	①No or small	56.85 ± 7.96	7.069	0.001	①②<③
	②Medium	57.65 ± 7.11			
	③Larger	61.21 ± 7.37			
Number of children	≤2	57.11 ± 7.75	−2.810	0.005	
	>2	59.83 ± 7.24			
Degree of disease understanding	①Very well	52.64 ± 8.72	6.023	0.003	①<②③
	②General	58.23 ± 7.11			
	③ Do not know	59.91 ± 7.72			

### 3.5. Factors influencing anxiety in patients with acute ischaemic stroke in the post-epidemic era

The regression equation was: anxiety’ Y = 27.918 + 6.242 × age − 9.123 × work status +0.274 × disease uncertainty +0.144 × acute stress response. The coefficient of determination *R*^2^ = 0.401, the coefficient of adjustment *R*^2^ = 0.356, and the ANOVA results with *F* = 8.760 and *p* = 0.000 indicate that the four included independent variables were able to explain 40.1% of the variance in anxiety symptoms of the study subjects, as detailed in Table 10.

**Table 10:** Regression analysis of anxiety in patients with acute ischaemic stroke (*n* = 240).

Independent Variables	Group	*β*	*SE*	*Beta*	*t*	*P*
Constant		27.918	5.400		5.170	<0.001
Age (years)	60 ~ 70	2.465	1.192	0.156	2.067	0.040
	≥70	3.777	1.254	0.236	3.013	0.003
Work status	Retired	−4.232	1.538	−0.273	−2.751	0.006
	Unemployed	−4.891	2.136	−0.282	−2.290	0.023
Sickness uncertainty		0.274	0.067	0.248	4.079	<0.001
Acute stress reaction		0.144	0.022	0.385	6.408	<0.001

*R* = 0.634, *R*^2^ = 0.401, adjusted *R*^2^ = 0.356.

### 3.6. Mediating effect test for acute stress response in the post-epidemic era

**Figure 3:**
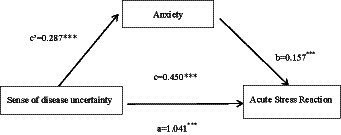
Intermediary effect model.

The results of the analysis showed that acute stress response partially mediated the prediction of anxiety by illness uncertainty. The variance of anxiety explained by acute stress response was sqrt (0.320–0.167) = 0.391 (39.1%), and the contribution of the mediating effect to the total effect was: Effect M = ab/c = 1.041 × 0.157/0.450 = 0.363 (36.3%), and the details are shown in Table 11 and Figure 3.

**Table 11:** Regression analysis of the mediating effect model of acute stress response.

Predictive variables	Model 1		Model 2		Model 3	
	*β*	*t*	*β*	*t*	*β*	*t*
Sense of disease uncertainty	1.041	5.828^***^	0.450	6.900^***^	0.287	4.546^***^
Acute stress response					0.157	7.318^***^
*R^2^*	0.125		0.167		0.320	
*F*	33.961^***^		47.604^***^		55.835^***^	

^**^p < 0.01. Model 1: Illness uncertainty predicts acute stress response; Model 2: Illness uncertainty predicts anxiety; Model 3: Illness uncertainty and acute stress response together predict anxiety.

## 4. Discussion

### 4.1. Analysis of factors affecting the sense of uncertainty about disease in the post-epidemic era

The more severe the sense of disease uncertainty was in patients over 60 years old, rural health insurance, no spouse, ethnic minority, and no knowledge of the disease. This is basically consistent with the findings of Wang Xiaoxiao (30–33) and others. Patients with high education had greater confidence in curing the disease, which is basically consistent with the findings of Wang Qiuling (34). Those who were employed scored significantly lower in disease uncertainty than those who were unemployed, which was inconsistent with the findings of foreign studies (35, 36). We hypothesise that this may be due to the fact that our social system, medical system, and cultural background are different from the situation of foreign patients. Patients with high *per capita* monthly household income are also better able to cope with acute ischaemic stroke and disability, which is largely similar to the results reported by Ni (37) and Kim et al. (38).

### 4.2. Analysis of factors influencing acute stress in the post-epidemic era

Older, spouseless patients, with no one to support them emotionally and spiritually and with only one person to support them financially, can develop a strong sense of isolation, triggering acute stress reactions, which is consistent with the findings of Wu Wei et al. (39). Highly educated and working patients can adapt to stressful events in a timely manner, which is generally consistent with the results reported by Suliman (40) and Song Qiong et al. (41). However, it is not consistent with the findings of Pingping Liu (42). Patients who are self-paying and self-assessed as financially stressed need to pay high medical costs in full and experience strong acute stress reactions in the face of unknown disease progression, which is generally consistent with the findings of Song Qiong et al. (43, 44). Therefore, on the issue of family support, during the hospitalisation of patients without spouses, healthcare workers should carry out more communication and exchanges to alleviate patients’ anxiety and loneliness, while relevant outdoor activities can be carried out in the community after discharge to enrich life. In terms of economic problems, it is recommended to increase the reimbursement ratio of medical insurance for sick families, and hospitals can carry out social donations for patients in critical condition and family difficulties to help them solve their economic pressure.

### 4.3. Analysis of the factors influencing anxiety in the post-epidemic era

The incidence of anxiety was significantly higher in patients who were older, had two or more children, were self-paying, had rural health insurance, had low education, and did not understand the disease (45–51). We hypothesise that this may be due to the fact that the number of children is an important measure of the patient’s emotional and spiritual support situation, that children may be in contentious conflict over the cost and responsibility of treatment, and that patients feel a heavy burden on their children.

### 4.4. Correlation analysis of sickness uncertainty, acute stress reaction and anxiety in the post-epidemic era

Sickness uncertainty is positively correlated with post-traumatic stress disorder, which is consistent with related scholars’ studies (52–57), and sickness uncertainty is a risk factor for the occurrence of post-traumatic stress disorder (55–58). There was a positive correlation between illness uncertainty and anxiety, which is consistent with the findings of Wong et al. (59). There was a positive correlation between acute stress response and anxiety, which is consistent with the findings of Meixiang Yin (60) and others.

### 4.5. Key influences on anxiety in the post-epidemic era

Age is an influencing factor for patients’ anxiety, and we hypothesise that it may be due to the decreased psychological flexibility of the elderly and the accumulated physical and mental burdens that aggravate their feelings of loneliness and helplessness. The inability to adapt to a new social role after retirement and a severe sense of loss may trigger other physical illnesses. Failure to understand the development and regression of the disease, resulting in negative psychological anxiety. Stimulating the organism to produce stress mechanisms, patients have strong stress reactions and anxiety due to unacceptability and difficulty in adapting.

### 4.6. The mediating role of anxiety in the post-epidemic era

The acute stress response partially mediates the prediction of anxiety by disease uncertainty, with the acute stress response explaining 39.1% of the variance in anxiety and the mediating effect contributing 36.3% of the total effect. When patients have illness uncertainty, different levels of acute stress responses affect patients’ anxiety levels, acute stress responses positively predict patients’ anxiety symptoms, and it was found that the stronger the patients’ illness uncertainty, the more pronounced the acute stress responses, thus aggravating patients’ anxiety to some extent, which is basically consistent with the findings of Hammen (61) on the predictive role of acute stress on anxiety. This study is based on the principles of the 2018 CANMAT/ISBD Guidelines for the Management of Bipolar Disorder published in March 2018 in Bipolar Disorders, the official journal of the ISBD, which is a major and comprehensive guideline update and revision after the 2013 edition of the Guidelines, which comprehensively evaluates the evidence-based evidence that has been added in recent years, and proposes new clinical recommendations, based on the full co-operation between CANMAT and the ISBD. Reduce anxiety in patients already burdened with stroke based on this guideline. Therefore, medical and nursing staff should focus on the psychological state of patients, actively guide patients to express their disease uncertainty, provide timely relief and excretion of acute stress reactions generated by patients, and enhance patients’ confidence to achieve the goal of reducing patients’ psychological burden and relieving anxiety.

The main hypothesis of the present study was confirmed, There was a positive correlation between anxiety and illness uncertainty, a positive correlation between anxiety and acute stress response, a positive correlation between illness uncertainty and acute stress response, and a partial mediating role of anxiety in illness uncertainty and acute stress response. There is such a relationship as illness uncertainty → anxiety → acute stress response.

## Data availability statement

The original contributions presented in the study are included in the article/supplementary material, further inquiries can be directed to the corresponding author.

## Ethics statement

Ethical approval was not required for the study involving humans in accordance with the local legislation and institutional requirements. Written informed consent to participate in this study was not required from the participants or the participants’ legal guardians/next of kin in accordance with the national legislation and the institutional requirements.

## Author contributions

HT is responsible for literature review, data collection, data statistics, and writing.
